# Cardiovascular risk of gabapentin and pregabalin in patients with diabetic neuropathy

**DOI:** 10.1186/s12933-022-01610-9

**Published:** 2022-09-01

**Authors:** Yiheng Pan, Pamela B. Davis, David C. Kaebler, Robert P. Blankfield, Rong Xu

**Affiliations:** 1 Center for Artificial Intelligence in Drug Discovery, Case Western Reserve University School of Medicine, Cleveland, OH USA; 2 Center for Community Health Integration, Case Western Reserve University School of Medicine, Cleveland, OH USA; 3 The Center for Clinical Informatics Research and Education, The MetroHealth System, Cleveland, OH USA; 4 Department of Family Medicine, Case Western Reserve University School of Medicine, Cleveland, OH USA

**Keywords:** Adverse cardiovascular events, Gabapentin, Pregabalin, Diabetic neuropathy, Cohort study

## Abstract

**Background:**

Gabapentin and pregabalin are commonly prescribed medications to treat pain in patients with diabetic neuropathy. Gabapentin and pregabalin can cause fluid retention, which is hypothesized to be associated with cardiovascular diseases. However, whether long-term use of gabapentin and pregabalin is associated with adverse cardiovascular diseases remains unknown. This study aims to examine the association between gabapentin use, pregabalin use and several adverse cardiovascular events.

**Methods:**

This retrospective cohort study used propensity score matching within patient electronic health records (EHRs) from a multicenter database with 106 million patients from 69 health care organizations in the US. The study population comprised 210,064 patients who had a diagnosis of diabetic neuropathy and were prescribed diabetic neuropathy medications in their EHRs. The exposure cohort comprised patients who were prescribed gabapentin or pregabalin to treat diabetic neuropathy. The comparison cohort comprised patients who were not prescribed either gabapentin or pregabalin but were prescribed other drugs to treat diabetic neuropathy. The outcomes of interest were myocardial infarcts, strokes, heart failure, peripheral vascular disease, and venous thromboembolic events. We calculated hazard ratios (HRs) and 95% confidence intervals (CIs) for 3-month and 5-year risk for adverse cardiovascular events between the propensity score-matched cohorts.

**Results:**

Both gabapentin and pregabalin were associated with increased risk of 5-year adverse cardiovascular events compared with the comparison group. In patients prescribed gabapentin, the highest risk was observed for deep venous thrombosis (HR: 1.58, 95% CI 1.37–1.82), followed by pulmonary embolism (HR: 1.5, 95% CI 1.27–1.76), peripheral vascular disease (HR: 1.37, 95% CI 1.27–1.47), stroke (HR: 1.31, 95% CI 1.2–1.43), myocardial infarction (HR: 1.25, 95% CI 1.14–1.38) and heart failure (HR: 1.14, 95% CI 1.07–1.21). In patients prescribed pregabalin, the highest risk was observed for deep venous thrombosis (HR: 1.57, 95% CI 1.31–1.88), followed by peripheral vascular disease (HR: 1.35, 95% CI 1.22–1.49), myocardial infarction (HR: 1.29, 95% CI 1.13–1.47), pulmonary embolism (HR: 1.28, 95% CI 1.04–1.59), stroke (HR: 1.26, 95% CI 1.12–1.42), and heart failure (HR: 1.2, 95% CI 1.11–1.3). There were significant associations between short-term (3 month) gabapentin use and heart failure, myocardial infarction, peripheral vascular disease, deep venous thrombosis, and pulmonary embolism. Short-term (3 month) pregabalin use was associated with deep venous thrombosis, peripheral vascular disease.

**Conclusion:**

In patients with diabetic neuropathy who were prescribed gabapentin and pregabalin, there is an increased risk for heart failure, myocardial infarction, peripheral vascular disease, stroke, deep venous thrombosis, and pulmonary embolism with long-term use. Our findings suggest that increased risk for adverse cardiovascular events, along with other side effects, the efficacy of pain control and the degree of tolerance of the patient, should be considered when prescribing gabapentin and pregabalin long-term in patients with diabetic neuropathy.

**Supplementary Information:**

The online version contains supplementary material available at 10.1186/s12933-022-01610-9.

## Introduction

The American Diabetes Association (ADA) and the European Association for the Study of Diabetes (EASD) provide annual guidance for clinicians. The standard of medical care in 2022 highlights cardiovascular disease management in patients with diabetes [1], as well as management of complications of diabetes, including diabetic neuropathy [2]. Neuropathic pain in diabetes can be severe and can impact quality of life [2]. Pregabalin and gabapentin are recommended as pharmacologic treatments for diabetic neuropathy [2]. While both medications were initially developed as anticonvulsants, pregabalin was approved to treat diabetic neuropathy in 2014 and gabapentin is often prescribed “off label” to treat diabetic neuropathy [3].

Both gabapentin and pregabalin can cause fluid retention by altering arterial myogenic tone [4]. Although fluid retention is not a recognized risk factor for cardiovascular disease, every medical condition that causes fluid retention—heart failure, nephrotic syndrome, cirrhosis, hypothyroidism, obstructive sleep apnea and Cushing’s syndrome—is associated with an increased risk of myocardial infarcts, strokes, and venous thromboembolic disease [5–19]. Moreover, previous studies have demonstrated that a number of medications that cause fluid retention are associated with an increased risk of adverse cardiovascular events. The list includes cyclo-oxygenase-2 inhibitors (COX-2 inhibitors) [20–23], nonselective non-steroidal anti-inflammatory drugs (NSAIDs) [22, 24, 25], hormone replacement therapy with estrogens and progestins [26, 27], oral contraceptives[28], insulins[29] and sulfonylureas [30, 31].

The consensus from multiple guidelines and systematic reviews is that gabapentinoids, serotonin norepinephrine reuptake inhibitors and tricyclic antidepressants have the best evidence to support their use in the treatment of diabetic neuropathic pain but comparative effectiveness studies to inform the best choice are lacking [32]. Opioid analgesics should be avoided due to their serious adverse effects and potential for addiction [3]. Long-term use of gabapentin and pregabalin is generally well tolerated and provides sustained efficacy [33, 34]. However, currently it remains unknown whether long-term use of gabapentin and pregabalin in patients with diabetic neuropathy is associated with increased risk for adverse cardiovascular events, including myocardial infarcts, strokes, heart failure, peripheral vascular disease, and venous thromboembolic events.

## Methods

### Database description

TriNetX research network is a de-identified population-level database with 106 million patients from 69 health care organizations, mostly large academic medical institutions with both inpatient and outpatient facilities at multiple locations across 50 states in the US, covering diverse geographic locations, age groups, racial and ethnic groups, income levels and insurance types [35]. Built-in statistical functions within TriNetX Analytics Platform perform statistical analyses on patient-level data and reports population level data and results without including protected health information (PHI) identifiers. MetroHealth System, Cleveland OH, Institutional Review Board (IRB) has determined that any research using TriNetX is not Human Subject Research and therefore exempt from IRB review.

### Study population

The study population comprised of 210,064 patients who had a diagnosis of diabetic neuropathy and were prescribed with diabetic neuropathy medications in their EHRs. The study population was divided into two cohorts: (1) an Exposure cohort consisting of patients who were prescribed gabapentin or pregabalin to treat diabetic neuropathy (International Classification of Diseases (ICD-10) diagnosis code: E11.40 Type 2 diabetes mellitus with diabetic neuropathy, unspecified) and (2) a Comparison cohort consisting of patients who were not prescribed either gabapentin or pregabalin but were prescribed other commonly-prescribed drugs (topiramate, duloxetine, tapentadol, capsaicin, nortriptyline, carbamazepine, venlafaxine, amitriptyline, and mexiletine) to control diabetic neuropathy [36, 37]. Patients who took no prescription medication for diabetic neuropathy were excluded since they were likely to have relatively mild diabetic neuropathy. We controlled for severity of diabetic neuropathy by including only patients in both the exposure cohort and the comparison cohort who were taking at least one prescription medication for diabetic neuropathy. To investigate the long-term effect, the long-term users were defined with the additional requirement that they had another drug prescription recorded more than 3 years after the first prescription.

### Statistical analysis

#### Outcome measures

The outcomes of interest were diagnoses of cardiovascular diseases including heart failure (ICD-10: I50 Heart failure), myocardial infarction (ICD-10: I21 Acute myocardial infarction), peripheral vascular disease (ICD-10: I73.9 Peripheral vascular diseases, unspecified), stroke (ICD-10: I63 Cerebral infarction), deep venous thrombosis (ICD-10: I82.40 Acute embolism and thrombosis of unspecified deep veins of lower extremity), and pulmonary embolism (ICD-10: I26 Pulmonary embolism).

#### Covariates

Covariates included demographics (age, gender, race, and ethnicity), risk factors of cardiovascular disease, initial drug indications including approved, and off-label uses, related medications, adverse socioeconomic circumstances, and specific risk factors for each disease.

The risk factors of cardiovascular diseases were hypertension (ICD-10: I10-I16 Hypertensive diseases), high cholesterol (ICD-10: E78.0 Pure hypercholesterolemia), obesity (ICD-10: E66 Overweight and obesity), diabetes (ICD-10: E08-E13 Diabetes mellitus), alcohol abuse (ICD-10: F10.1 Alcohol abuse), tobacco use (ICD-10: Z72.0 Tobacco use), use of NSAID (ICD-10: Z79.1 long-term (current) use of non-steroidal anti-inflammatories), obstructive sleep apnea [ICD-10: G47.33 Obstructive sleep apnea (adult) (pediatric)], end stage renal disease (ICD-10: N18.6 End stage renal disease), pre-existing heart failure (ICD-10: I50 Heart failure) and pre-existing peripheral vascular disease (ICD-10: I73.9 Peripheral vascular diseases, unspecified). Adverse socioeconomic circumstances were determined by incorporating ICD-10: Z55-Z65 Persons with potential health hazards related to socioeconomic and psychosocial circumstances. The medication list, presented in Additional file 1: Table S2, includes the medications for diabetic neuropathy, medications that can cause fluid retention and medications that can raise the blood pressure.

The initial indications were diabetic neuropathy (ICD-10: E11.40 Type 2 diabetes mellitus with diabetic neuropathy, unspecified), seizure (ICD-10: G40 Epilepsy and recurrent seizures), postherpetic neuralgia (ICD-10: B02.29 Other postherpetic nervous system involvement), fibromyalgia (ICD-10: M79.7 Fibromyalgia), pain (ICD-10: R52 Pain, unspecified), neuropathic pain (ICD-10: M79.2 Neuralgia and neuritis, unspecified), restless leg syndrome (ICD-10: G25.81 Restless leg syndrome).

The specific risk factors and complications for heart failure (ICD-10: I50 Heart failure) were myocardial infarction (ICD-10: I21 Acute myocardial infarction), myocarditis (ICD-10: I51.4 Myocarditis, unspecified), and arrhythmias (ICD-10: I49 Other cardiac arrhythmias); for myocardial Infarction (ICD-10: I21 Acute myocardial infarction) were sudden cardiac arrest (ICD-10: Z86.74 Personal history of sudden cardiac arrest), and metabolic syndrome (ICD-10: E88.81 Metabolic syndrome); for stroke (ICD-10: I63 Cerebral infarction) were atrial fibrillation (ICD-10: I48 Atrial fibrillation and flutter), arrhythmias (ICD-10: I49 Other cardiac arrhythmias), and depression (ICD-10: F32 Depressive episode), for peripheral vascular disease (ICD-10: I73.9 Peripheral vascular diseases, unspecified) were stroke (ICD-10: I63 Cerebral infarction), myocardial infarction (ICD-10: I21 Acute myocardial infarction), and atherosclerosis (ICD-10: I70 Atherosclerosis); for deep venous thrombosis (ICD-10: I82.40 Acute embolism and thrombosis of unspecified deep veins of lower extremity) were inflammatory bowel disease (ICD-10: K51.9 Ulcerative colitis, unspecified), and cancer (ICD-10: C80.1 Malignant (primary) neoplasm, unspecified); for pulmonary embolism (ICD-10: I26 Pulmonary embolism) was cancer (ICD-10: C80.1 Malignant (primary) neoplasm, unspecified). The full list of covariates, their standardized names, codes, and data types in TriNetX are described in Additional file 1: Table S2.

The Exposure cohort and Comparison cohort were propensity-score matched (1:1 matching using the nearest neighbor greedy matching algorithm) for covariates described above. Kaplan–Meier analysis was performed to estimate the risk of each cardiovascular adverse event in the Exposure and Comparison cohorts. The time index for the exposure and the comparison is the drug first prescription date. 3-month outcomes and 5-year outcomes are determined by following patients with studied outcomes within 3-month and 5-year time frame since the drug prescription. Hazard ratios, 95% confidence interval and p-value were calculated at significance set at p-value < 0.05 (two-sided).

## Results

### Patient characteristics

Table 1 and Additional file 1: Table S3 presents characteristics of the gabapentin and comparison groups for long-term use before and after propensity score matching for covariates of deep venous thrombosis. Data for pregabalin and patient characteristics for short-term drug use are in the Supplement (Additional file 1: Table S4–S6). Patients with diabetic neuropathy who were prescribed gabapentin were younger than those prescribed comparison drugs (60.5 versus 62.8), comprised fewer women, more African Americans, Hispanics and had fewer comorbidities. After propensity-score matching, the two cohorts were balanced.

**Table 1:** The characteristics of the patients who were prescribed gabapentin and comparison drugs for long term before and after propensity-score matching for covariates of deep venous thrombosis

Characteristics	Before matching			After matching		
	Cohort, No. (%)			Cohort, No. (%)		
	Gabapentin	Comparison	SMD	Gabapentin	Comparison	SMD
Cohort size	38,603	8359		7050	7050	
Age at Index	60.5 ± 12.3	62.8 ± 12.5	0.19*	61.5 ± 11.7	62.2 ± 12.5	0.06
Female	54.0	61.7	0.16*	63.5	61.6	0.04
Race and ethnicity						
White	64.7	72.7	0.17*	70.9	71.7	0.02
African American	26.5	16.4	0.25*	19.4	17.6	0.05
Unknown Race	7.0	9.5	0.09	8.5	9.3	0.03
Hispanic or Latino	9.5	5.9	0.13*	6.4	6.5	0.01
Asian	1.2	0.8	0.04	0.7	0.8	0.01
Cardiovascular risk factors						
Hypertensive diseases	85.0	87.4	0.07	86.9	87.1	0.01
Obesity	40.1	47.2	0.14*	47.3	47.1	0.00
High cholesterol	23.1	28.6	0.13*	28.0	28.1	0.00
Obstructive sleep apnea	18.0	24.5	0.16*	24.4	23.9	0.01
Tobacco use	4.1	6.7	0.11*	5.9	6.5	0.03
Pre-existing heart failure	17.8	20.0	0.06	19.1	20.1	0.02
Pre-existing peripheral vascular disease	14.4	14.3	0.00	14.3	14.1	0.01
End stage renal disease	4.5	4.3	0.01	3.8	4.2	0.02
Alcohol abuse	4.0	3.7	0.01	4.1	3.8	0.01
Long term use of NSAID	1.4	2.6	0.09	2.0	2.3	0.02
Adverse socioeconomic circumstances	4.6	7.6	0.12*	6.7	7.5	0.03
Initial use						
Postherpetic neuralgia	0.6	0.4	0.03	0.5	0.5	0.01
Fibromyalgia	11.7	14.0	0.07	15.7	14.6	0.03
Pain	9.5	11.7	0.07	11.2	11.8	0.02
Restless legs syndrome	4.4	4.9	0.03	5.5	5.0	0.02
Epilepsy and recurrent seizures	2.7	6.3	0.17*	5.3	5.7	0.02
Neuralgia and neuritis, unspecified	4.7	4.1	0.03	4.5	4.3	0.01
Specific risk factor of deep venous thrombosis						
Pre-existing deep venous thrombosis	2.9	3.6	0.04	3.4	3.5	0.01
Cancer	1.2	2.0	0.07	1.8	2.0	0.01
Inflammatory bowel disease	0.4	0.6	0.03	0.5	0.6	0.02

*SMD* standardized mean differences

*SMD greater than 0.1, a threshold being recommended for declaring imbalance

### Risk of 5-year adverse cardiovascular events associated with gabapentin and pregabalin in patients with diabetic neuropathy

**Fig. 1:**
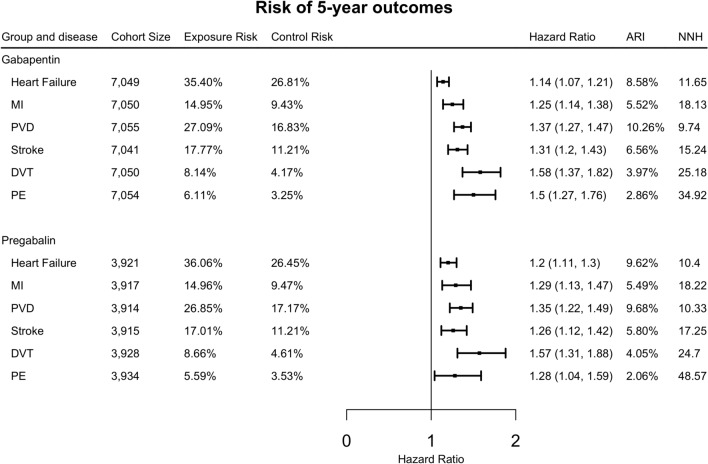
Risk of 5-year outcomes in propensity-score matched exposure groups and comparison groups in the patients who had taken the drugs long term. (ARI: absolute risk increase, NNH: number need to harm, MI: myocardial infarction, PVD: peripheral vascular disease, DVT: deep venous thrombosis, PE: pulmonary embolism)

As shown in Fig. 1, both gabapentin and pregabalin were associated with increased risk of 5-year adverse cardiovascular events compared with the comparison group. In patients prescribed gabapentin, the highest risk was observed for deep venous thrombosis (HR: 1.58, 95% CI 1.37–1.82), followed by pulmonary embolism (HR: 1.5, 95% CI 1.27–1.76), peripheral vascular disease (HR: 1.37, 95% CI 1.27–1.47), stroke (HR: 1.31, 95% CI 1.2–1.43), myocardial infarction (HR: 1.25, 95% CI 1.14–1.38) and heart failure (HR: 1.14, 95% CI 1.07–1.21). In patients prescribed pregabalin, the highest risk was observed for deep venous thrombosis (HR: 1.57, 95% CI 1.31–1.88), followed by peripheral vascular disease (HR: 1.35, 95% CI 1.22–1.49), myocardial infarction (HR: 1.29, 95% CI 1.13–1.47), pulmonary embolism (HR: 1.28, 95% CI 1.04–1.59), stroke (HR: 1.26, 95% CI 1.12–1.42), and heart failure (HR: 1.2, 95% CI 1.11–1.3).

### Risk of 3-month adverse cardiovascular events associated with gabapentin and pregabalin in patients with diabetic neuropathy

**Fig. 2:**
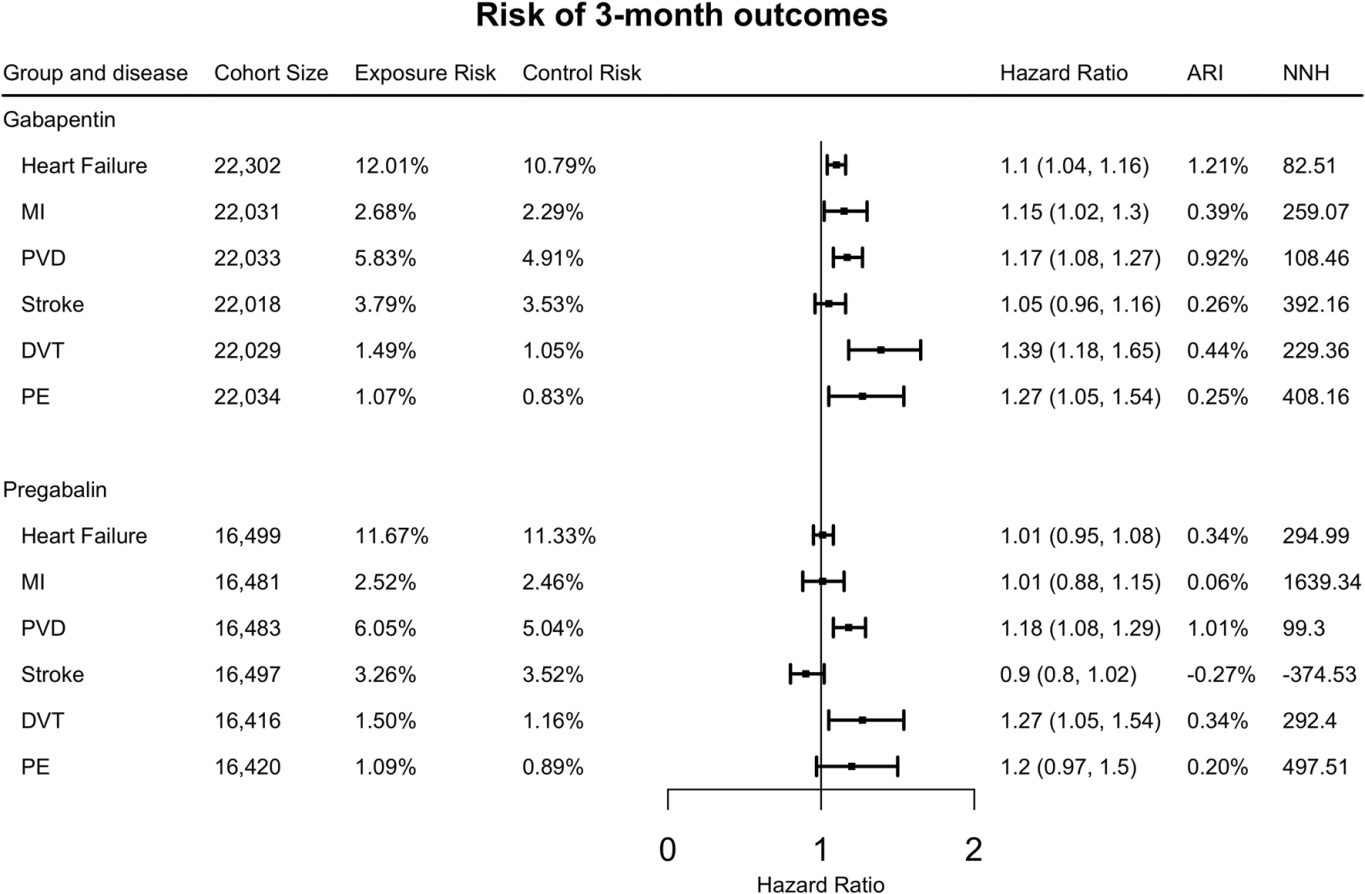
Risk of 3-month outcomes in propensity-score matched exposure groups and comparison groups in the patients who had ever taken the drugs. (ARI: absolute risk increase, NNH: number need to harm, MI: myocardial infarction, PVD: peripheral vascular disease, DVT: deep venous thrombosis, PE: pulmonary embolism)

As shown in Fig. 2, gabapentin was associated with an increased risk of deep venous thrombosis (HR: 1.39, 95% CI 1.18–1.65), pulmonary embolism (HR: 1.27, 95% CI 1.05–1.54), peripheral vascular disease (HR: 1.17, 95% CI 1.08–1.27), myocardial infarction (HR: 1.15, 95% CI 1.02–1.3), and heart failure (HR: 1.1, 95% CI 1.04–1.16) compared with the comparison group within the 3-month time frame following the drug treatment. There was no significant association between gabapentin and risk of stroke (HR: 1.05, 95% CI 0.96–1.16). Pregabalin was associated with an increased risk of 3-month adverse cardiovascular events for deep venous thrombosis (HR: 1.27, 95% CI 1.05–1.54), peripheral vascular disease (HR: 1.18, 95% CI 1.08–1.29). There was no association between pregabalin and risk of 3-month adverse cardiovascular events for pulmonary embolism (HR: 1.2, 95% CI 0.97–1.5), myocardial infarction (HR: 1.01, 95% CI 0.88–1.15), stroke (HR: 0.9, 95% CI 0.8–1.02), and heart failure (HR: 1.01, 95% CI 0.95–1.08).

## Discussion

Using the large-scale nation-wide database of patient de-identified TriNetX EHRs, our study found that there is an increased risk of adverse cardiovascular events, including heart failure, myocardial infarction, peripheral vascular disease, stroke, deep venous thrombosis, and pulmonary embolism in patients with diabetic neuropathy, following long-term use of gabapentin and pregabalin, with highest risk for deep venous thrombosis. There were significant associations between short-term (3 month) gabapentin use and heart failure, myocardial infarction, peripheral vascular disease, deep venous thrombosis, and pulmonary embolism. Short-term (3 month) pregabalin use was associated with deep venous thrombosis and peripheral vascular disease.

Associations between gabapentin, pregabalin and heart failure have been reported in case reports [38, 39], but not in observational studies [40, 41]. These case reports and observational studies are limited to short-term follow up [38–41]. To the best of our knowledge, this study is among the first to report that long-term use of gabapentin and pregabalin is associated with increased risk for adverse cardiovascular events in patients with diabetic neuropathy. While the cardiovascular effects of gabapentin and pregabalin need to be verified in other populations that use these drugs, our findings call for thoughtful consideration of risk and benefit when deciding to use gabapentin and pregabalin long-term in patients with diabetic neuropathy, and possibly in other populations as well.

One possible explanation for the findings is that gabapentin and pregabalin can alter arterial myogenic tone and cause fluid retention [4]. Fluid retention causes either an increase in cardiac output or an increase in blood pressure [42]. Velocity of blood flow increases either way, thereby increasing turbulence of blood flow [43]. Increased turbulence reduces shear stress upon arterial walls, thereby increasing endothelial dysfunction, which is a well-established cardiovascular risk factor [44]. However, our finding cannot rule out other potential mechanisms. Future research is necessary to further understand the underlying mechanism of the observed increased risk of CVDs among patients with diabetic neuropathy who were prescribed gabapentin and pregabalin. Additional work is needed to determine if other medications that cause fluid retention are also associated with increased adverse cardiovascular events. Anti-hypertensive medications that cause fluid retention, notably calcium channel blockers, alpha receptor antagonists and hydralazine, have favorable cardiovascular effects. Aromatase inhibitors and androgen deprivation therapy cause fluid retention but increase the longevity of cancer patients. For medications that do not improve longevity, do not lower blood pressure (BP) and that are used long-term, such as androgens, corticosteroids (when used to treat non-life-threatening conditions), mineralocorticoids, pramipexole and ropinirole, patients and clinicians need to know if there is an increased risk of cardiovascular disease. For androgens, the research literature is inconclusive concerning cardiovascular risk: some studies have documented an increased risk of adverse cardiovascular events while other studies have failed to do so [45, 46]. Cardiovascular safety data is needed for medications that cause fluid retention but do not increase longevity or lower BP.

If an increased risk of adverse cardiovascular events is also associated with other medications that cause fluid retention, then our study has implications for the pharmaceutical industry and for the entities responsible for regulating pharmaceutical products: the Food and Drug Administration in the United States, the European Medicines Agency in the European Union, the Medicines and Healthcare Products Regulatory Agency in Britain, and the Health Products and Food Branch of Health Canada. If fluid retention is a risk factor for cardiovascular disease, then data regarding the fluid retention properties of medications would be relevant to the drug approval process. In that circumstance, for medications that do not extend life, do not lower BP and are intended to be used long term, regulatory agencies should require new drug applications to provide fluid retention data. Cardiovascular safety data would be warranted for medications that cause fluid retention.

Many patients take medications that cause fluid retention. One implication of our study is that it may be possible to mitigate the cardiovascular consequences of medications that cause fluid retention, at least in part, by simultaneously prescribing a diuretic. This is a potential area for future research.

Numerous risk factors for cardiovascular diseases (CVDs) have been recognized, including age, gender, smoking status, hypertension, diabetes, hypercholesterolemia, physical inactivity, obstructive sleep apnea and overweight [16, 47]. Findings from this study suggest that long-term use of gabapentin/pregabalin is another risk factor for CVDs in patients with diabetic neuropathy. Future research is needed to understand whether these risk factors act synergistically or independently in increasing the risk for CVDs in specific patient populations.

Several limitations of this study should be noted. First, the patients in the TriNetX EHR database represent people who had medical encounters with the 69 contributing health care systems. The generalizability of the results needs to be validated in other national data resources. Second, there is no data linkage between the drug prescriptions and diagnosis codes. We assumed that if the diagnosis of diabetic neuropathy happened before the drug prescription, then the patients were taking the drug for treating diabetic neuropathy. Nonetheless, there might be other indications for prescribing these drugs. We addressed this problem by controlling the other initial uses in propensity score matching. Third, to control for severity of diabetic neuropathy, we excluded patients not taking medications for treatment of diabetic neuropathy since they might have relatively mild diabetic neuropathy. The control cohort included patients taking other medications to treat diabetic neuropathy. The exposure group could also take other medications to treat diabetic neuropathy, in addition to gabapentin and pregabalin (“polypharmacy”). For propensity-score matching, the exposure and control cohorts were matched for specific drugs that are commonly prescribed to treat diabetic neuropathy. Fourth, due to the nature of EHR data, TriNetX platform captures patient information that is entered during patients’ encounter with healthcare organization. Consequently, there is no guarantee of completeness for specific research purposes. For example, other events (e.g., diet, certain lifestyle factors, employment) that happened outside of patient medical encounters may not be captured in patient EHRs. However, because we compared exposure and control cohorts all from TriNetX database, selection bias should not substantially affect the relative risk analyses. Though the exposure and control cohorts were extensively propensity-score matched for demographics, original disease indications, risk factors, and other commonly prescribed medications, we acknowledge the limitation of unmeasured confounders that are not included in patient EHRs. Fifth, drug duration was an important covariate. We used a computerized database of medical information to define exposure to gabapentin and pregabalin. The database identifies only that the medications were prescribed, not that they were consumed, and electronic health records cannot provide information about how long patients actually take any given drug. Nonetheless, automated pharmacy records are generally reliable sources of information on drug use [48, 49]. We addressed this problem by defining long-term users with an additional query for the patients who have another drug prescription recorded more than 3 years after the first drug prescription. Sixth, we could not determine the doses of gabapentin or pregabalin that were prescribed. It is possible that the cardiovascular risk of these medications is dose dependent such that low doses may not be associated with an increased risk of adverse cardiovascular events.

## Conclusion

We identified an increased risk of adverse cardiovascular events associated with use of gabapentin and pregabalin in patients with diabetic neuropathy. Our findings need to be verified in other populations of gabapentin and pregabalin users and with other medications that cause fluid retention. If our results are generalizable, increased risk of adverse cardiovascular outcomes, along with other side effects, the efficacy of pain control and the degree of tolerance of the patient, should inform the decision-making process when prescribing gabapentin, pregabalin, and perhaps other medications that cause fluid retention.

## Supplementary Information

**Additional file 1: Table S1.** Outcomes and their standardized names, codes and data types that are used in the TriNetX database. **Table S2.** Covariates and their standardized names, codes and data types that are used in the TriNetX database. **Table S3.** The characteristics of the patients who were prescribed gabapentin and comparison drugs for long term before and after propensity-score matching for covariates of deep venous thrombosis. **Table S4.** The characteristics of the patients who were prescribed pregabalin and comparison drugs for long term before and after propensity-score matching for covariates of deep venous thrombosis. **Table S5.** The characteristics of the patients who were prescribed gabapentin and comparison drugs before and after propensity-score matching for covariates of deep venous thrombosis. **Table S6.** The characteristics of the patients who were prescribed pregabalin and comparison drugs before and after propensity-score matching for covariates of deep venous thrombosis.

## Data Availability

The data that support the findings of this study are available from TriNetX [35] (https://trinetx.com/).

## Abbreviations

EHRs: Electronic health records
CVDs: Cardiovascular diseases
HRs: Hazard ratios
CIs: Confidence intervals
NSAIDs: Nonselective non-steroidal anti-inflammatory drugs
BP: Blood pressure
SMD: Standardized mean difference
