# Supplementary material for: Cardiovascular risk of gabapentin and pregabalin in patients with diabetic neuropathy
Source: Cardiovasc Diabetol. 2022 Sep 1;21:170. doi: 10.1186/s12933-022-01610-9 (PMC9438165; doi:10.1186/s12933-022-01610-9)
Supplement: Supplementary file 1 — Additional file 1: Table S1. Outcomes and their standardized names, codes and data types that are used in the TriNetX database. Table S2. Covariates and their standardized names, codes and data types that are used in the TriNetX database. Table S3. The characteristics of the patients who were prescribed gabapentin and comparison drugs for long term before and after propensity-score matching for covariates of deep venous thrombosis. Table S4. The characteristics of the patients who were prescribed pregabalin and comparison drugs for long term before and after propensity-score matching for covariates of deep venous thrombosis. Table S5. The characteristics of the patients who were prescribed gabapentin and comparison drugs before and after propensity-score matching for covariates of deep venous thrombosis. Table S6. The characteristics of the patients who were prescribed pregabalin and comparison drugs before and after propensity-score matching for covariates of deep venous thrombosis. [file 12933_2022_1610_MOESM1_ESM.docx]

Supplemental Information

Cardiovascular risk of gabapentin and pregabalin in patients with diabetic neuropathy

**S1 Table:** Outcomes and their standardized names, codes and data types that are used in the TriNetX database

**S2 Table:** Covariates and their standardized names, codes and data types that are used in the TriNetX database

**S3 Table:** The characteristics of the patients who were prescribed gabapentin and comparison drugs for long term before and after propensity-score matching for covariates of deep venous thrombosis.

**S4 Table:** The characteristics of the patients who were prescribed pregabalin and comparison drugs for long term before and after propensity-score matching for covariates of deep venous thrombosis.

**S5 Table:** The characteristics of the patients who were prescribed gabapentin and comparison drugs before and after propensity-score matching for covariates of deep venous thrombosis.

**S6 Table:** The characteristics of the patients who were prescribed pregabalin and comparison drugs before and after propensity-score matching for covariates of deep venous thrombosis.

**S1 Table:** Outcomes and their standardized names, codes and data types that are used in the TriNetX database

| **Outcomes** | **Code** | **Standardized names** | **Data type** |
| --- | --- | --- | --- |
| Heart failure | ICD-10: I50 | Heart failure | Present/absent |
| Myocardial infarction | ICD-10: I21 | Acute myocardial infarction | Present/absent |
| Peripheral vascular disease | ICD-10: I73.9 | Peripheral vascular diseases, unspecified | Present/absent |
| Stroke | ICD-10: I63 | Cerebral infarction | Present/absent |
| Deep venous thrombosis | ICD-10: I82.40 | Acute embolism and thrombosis of unspecified deep veins of lower extremity | Present/absent |
| Pulmonary embolism | ICD-10: I26 | Pulmonary embolism | Present/absent |

**S2 Table:** Covariates and their standardized names, codes and data types that are used in the TriNetX database

| **Covariate** | **Code** | **Standardized names** | **Data type** |
| --- | --- | --- | --- |
| Demographics | | | |
| Age at Index | AI | Age at Index | Continuous |
| Female | Demographics: F | Female | Present/absent |
| White | Demographics: 2106-3 | White | Present/absent |
| Black or African American | Demographics: 2054-5 | Black or African American | Present/absent |
| Unknown Race | Demographics: 2131-1 | Unknown Race | Present/absent |
| Hispanic or Latino | Demographics: 2135-2 | Hispanic or Latino | Present/absent |
| Asian | Demographics: 2028-9 | Asian | Present/absent |
| Comorbidities and risk factors | | | |
| Diabetes mellitus | ICD-10: E08-E13 | Diabetes mellitus | Present/absent |
| Hypertension | ICD-10: I10-I16 | Hypertensive diseases | Present/absent |
| Overweight and obesity | ICD-10: E66 | Overweight and obesity | Present/absent |
| High cholesterol | ICD-10: E78.0 | Pure hypercholesterolemia | Present/absent |
| Obstructive sleep apnea | ICD-10: G47.33 | Obstructive sleep apnea (adult) (pediatric) | Present/absent |
| Long term use of NSAID | ICD-10: Z79.1 | Long term (current) use of non-steroidal anti-inflammatories (NSAID) | Present/absent |
| End stage renal disease | ICD-10: N18.6 | End stage renal disease | Present/absent |
| Adverse socioeconomic determinants of health | ICD-10: Z55-Z65 | Persons with potential health hazards related to socioeconomic and psychosocial circumstances | Present/absent |
| Alcohol abuse | ICD-10: F10.1 | Alcohol abuse | Present/absent |
| Tobacco use | ICD-10: Z72.0 | Tobacco use | Present/absent |
| Pre-existing heart failure | ICD-10: I50 | Heart failure | Present/absent |
| Pre-existing peripheral vascular disease | ICD-10: I73.9 | Peripheral vascular diseases, unspecified | Present/absent |
| Initial indications | | | |
| Diabetic Neuropathy | ICD-10: E11.40 | Type 2 diabetes mellitus with diabetic neuropathy, unspecified | Present/absent |
| Seizure | ICD-10: G40 | Epilepsy and recurrent seizures | Present/absent |
| Fibromyalgia | ICD-10: M79.7 | Fibromyalgia | Present/absent |
| Pain | ICD-10: R52 | Pain, unspecified | Present/absent |
| Neuropathic pain | ICD-10: M79.2 | Neuralgia and neuritis, unspecified | Present/absent |
| Postherpetic neuralgia | ICD-10: B02.29 | Other postherpetic nervous system involvement | Present/absent |
| Restless leg syndrome | ICD-10: G25.81 | Restless legs syndrome | Present/absent |
| Risk factors of heart failure | | | |
| Pre-existing heart failure | ICD-10: I50 | Heart failure | Present/absent |
| Myocardial infarction | ICD-10: I21 | Acute myocardial infarction | Present/absent |
| Myocarditis | ICD-10: I51.4 | Myocarditis, unspecified | Present/absent |
| Arrhythmias | ICD-10: I49 | Other cardiac arrhythmias | Present/absent |
| Risk factors of myocardial infarction | | | |
| Pre-existing myocardial infarction | ICD-10: I21 | Acute myocardial infarction | Present/absent |
| Sudden cardiac arrest | ICD-10: Z86.74 | Personal history of sudden cardiac arrest | Present/absent |
| Metabolic syndrome | ICD-10: E88.81 | Metabolic syndrome | Present/absent |
| Risk factors of stroke | | | |
| Pre-existing stroke | ICD-10: I63 | Cerebral infarction | Present/absent |
| Atrial fibrillation | ICD-10: I48 | Atrial fibrillation and flutter | Present/absent |
| Arrhythmias | ICD-10: I49 | Other cardiac arrhythmias | Present/absent |
| Depression | ICD-10: F32 | Depressive episode | Present/absent |
| Risk factors of peripheral vascular disease | | | |
| Pre-existing peripheral vascular disease | ICD-10: I73.9 | Peripheral vascular diseases, unspecified | Present/absent |
| Stroke | ICD-10: I63 | Cerebral infarction | Present/absent |
| Myocardial infarction | ICD-10: I21 | Acute myocardial infarction | Present/absent |
| Atherosclerosis | ICD-10: I70 | Atherosclerosis | Present/absent |
| Risk factors of deep venous thrombosis | | | |
| Pre-existing deep venous thrombosis | ICD-10: I82.40 | Acute embolism and thrombosis of unspecified deep veins of lower extremity | Present/absent |
| Inflammatory bowel disease | ICD-10: K51.9 | Ulcerative colitis, unspecified | Present/absent |
| Cancer | ICD-10: C80.1 | Malignant (primary) neoplasm, unspecified | Present/absent |
| Risk factors of pulmonary embolism | | | |
| Pre-existing pulmonary embolism | ICD-10: I26 | Pulmonary embolism | Present/absent |
| Cancer | ICD-10: C80.1 | Malignant (primary) neoplasm, unspecified | Present/absent |
| Medications | | | |
| Cardiovascular medications | RxNorm: CV000 | CARDIOVASCULAR MEDICATIONS | Present/absent |
| Antilipemic agents | RxNorm: CV350 | ANTILIPEMIC AGENTS | Present/absent |
| Insulin | RxNorm: HS501 | INSULIN | Present/absent |
| Metformin | RxNorm: 6809 | metformin | Present/absent |
| Diuretics | RxNorm: CV700 | DIURETICS | Present/absent |
| Beta blockers | RxNorm: CV100 | BETA BLOCKERS/RELATED | Present/absent |
| Ace inhibitors | RxNorm: CV800 | ACE INHIBITORS | Present/absent |
| Aspirin | RxNorm: 1191 | aspirin | Present/absent |
| Hydrocodone | RxNorm: 5489 | hydrocodone | Present/absent |
| Antiarrhythmics | RxNorm: CV300 | ANTIARRHYTHMICS | Present/absent |
| Oxycodone | RxNorm: 7804 | oxycodone | Present/absent |
| Duloxetine | RxNorm: 72625 | duloxetine | Present/absent |
| Calcium channel blockers | RxNorm: CV200 | CALCIUM CHANNEL BLOCKERS | Present/absent |
| Antihypoglycemics | RxNorm: HS503 | ANTIHYPOGLYCEMICS | Present/absent |
| Amitriptyline | RxNorm: 704 | amitriptyline | Present/absent |
| Sulfonylureas | RxNorm: A10BB | Sulfonylureas | Present/absent |
| Tramadol | RxNorm: 10689 | tramadol | Present/absent |
| Iburofen | RxNorm: 5640 | ibuprofen | Present/absent |
| Angiotension ii inhibitor | RxNorm: CV805 | ANGIOTENSIN II INHIBITOR | Present/absent |
| Other antihypertensives | RxNorm: CV490 | ANTIHYPERTENSIVES,OTHER | Present/absent |
| Prednisone | RxNorm: 8640 | prednisone | Present/absent |
| Methylprednisolone | RxNorm: 6902 | methylprednisolone | Present/absent |
| Antianginals | RxNorm: CV250 | ANTIANGINALS | Present/absent |
| Codeine | RxNorm: 2670 | codeine | Present/absent |
| Naproxen | RxNorm: 7258 | naproxen | Present/absent |
| Antipsychotics | RxNorm: N05A | ANTIPSYCHOTICS | Present/absent |
| Venlafaxine | RxNorm: 39786 | venlafaxine | Present/absent |
| Diclofenac | RxNorm: 3355 | diclofenac | Present/absent |
| Alpha blockers | RxNorm: CV150 | ALPHA BLOCKERS/RELATED | Present/absent |
| Topiramate | RxNorm: 38404 | topiramate | Present/absent |
| Bupropion | RxNorm: 42347 | bupropion | Present/absent |
| Nortriptyline | RxNorm: 7531 | nortriptyline | Present/absent |
| Thiazolidinediones | RxNorm: A10BG | Thiazolidinediones | Present/absent |
| Celecoxib | RxNorm: 140587 | celecoxib | Present/absent |
| Other hypoglycemic agents | RxNorm: HS509 | HYPOGLYCEMIC AGENTS,OTHER | Present/absent |
| Estrogens | RxNorm: G03C | ESTROGENS | Present/absent |
| Capsaicin | RxNorm: 1992 | capsaicin | Present/absent |
| Other cardiovascular agents | RxNorm: CV900 | CARDIOVASCULAR AGENTS,OTHER | Present/absent |
| Antimigraine agents | RxNorm: CN105 | ANTIMIGRAINE AGENTS | Present/absent |
| Liraglutide | RxNorm: 475968 | liraglutide | Present/absent |
| Sodium-glucose co-transporter 2 inhibitors | RxNorm: A10BK | Sodium-glucose co-transporter 2 (SGLT2) inhibitors | Present/absent |
| Progestins | RxNorm: HS800 | PROGESTINS | Present/absent |
| Exenatide | RxNorm: 60548 | exenatide | Present/absent |
| Carbamazepine | RxNorm: 2002 | carbamazepine | Present/absent |
| Androgens | RxNorm: G03B | ANDROGENS | Present/absent |
| Dulaglutide | RxNorm: 1551291 | dulaglutide | Present/absent |
| Digitalis glycosides | RxNorm: CV050 | DIGITALIS GLYCOSIDES | Present/absent |
| Peripheral vasodilators | RxNorm: CV500 | PERIPHERAL VASODILATORS | Present/absent |
| Tapentadol | RxNorm: 787390 | tapentadol | Present/absent |
| Mineralocorticoids | RxNorm: H02AA | Mineralocorticoids | Present/absent |
| Semaglutide | RxNorm: 1991302 | semaglutide | Present/absent |
| Tamoxifen | RxNorm: 10324 | tamoxifen | Present/absent |
| Gonadotropin releasing hormone analogues | RxNorm: L02AE | Gonadotropin releasing hormone analogues | Present/absent |
| Antihypertensive combinations | RxNorm: CV400 | ANTIHYPERTENSIVE COMBINATIONS | Present/absent |
| Raloxifene | RxNorm: 72143 | raloxifene | Present/absent |
| Mexiletine | RxNorm: 6926 | mexiletine | Present/absent |
| Direct renin inhibitor | RxNorm: CV806 | DIRECT RENIN INHIBITOR | Present/absent |
| Sclerosing agents | RxNorm: CV600 | SCLEROSING AGENTS | Present/absent |
| Lixisenatide | RxNorm: 1440051 | lixisenatide | Present/absent |

**S3 Table:** The characteristics of the patients who were prescribed gabapentin and comparison drugs for long term before and after propensity-score matching for covariates of deep venous thrombosis. SMD – standardized mean differences. *SMD greater than 0.1, a threshold being recommended for declaring imbalance

|  | Before matching | | | After matching | | |
| --- | --- | --- | --- | --- | --- | --- |
|  | Cohort, No. (%) | | | Cohort, No. (%) | | |
|  | Gabapentin | Comparison | SMD | Gabapentin | Comparison | SMD |
| Cohort size | 38603 | 8359 |  | 7050 | 7050 |  |
| Age at Index | 60.5 ± 12.3 | 62.8 ± 12.5 | 0.19* | 61.5 ± 11.7 | 62.2 ± 12.5 | 0.06 |
| White | 64.7% | 72.7% | 0.17* | 70.9% | 71.7% | 0.02 |
| Female | 54.0% | 61.7% | 0.16* | 63.5% | 61.6% | 0.04 |
| Black or African American | 26.5% | 16.4% | 0.25* | 19.4% | 17.6% | 0.05 |
| Unknown Race | 7.0% | 9.5% | 0.09 | 8.5% | 9.3% | 0.03 |
| Hispanic or Latino | 9.5% | 5.9% | 0.13* | 6.4% | 6.5% | 0.01 |
| Asian | 1.2% | 0.8% | 0.04 | 0.7% | 0.8% | 0.01 |
| Hypertensive diseases | 85.0% | 87.4% | 0.07 | 86.9% | 87.1% | 0.01 |
| Overweight and obesity | 40.1% | 47.2% | 0.14* | 47.3% | 47.1% | 0.00 |
| Pure hypercholesterolemia | 23.1% | 28.6% | 0.13* | 28.0% | 28.1% | 0.00 |
| Obstructive sleep apnea (adult) (pediatric) | 18.0% | 24.5% | 0.16* | 24.4% | 23.9% | 0.01 |
| Heart failure | 17.8% | 20.0% | 0.06 | 19.1% | 20.1% | 0.02 |
| Fibromyalgia | 11.7% | 14.0% | 0.07 | 15.7% | 14.6% | 0.03 |
| Peripheral vascular disease, unspecified | 14.4% | 14.3% | 0.00 | 14.3% | 14.1% | 0.01 |
| Pain, unspecified | 9.5% | 11.7% | 0.07 | 11.2% | 11.8% | 0.02 |
| Persons with potential health hazards related to socioeconomic and psychosocial circumstances | 4.6% | 7.6% | 0.12* | 6.7% | 7.5% | 0.03 |
| Tobacco use | 4.1% | 6.7% | 0.11* | 5.9% | 6.5% | 0.03 |
| Restless legs syndrome | 4.4% | 4.9% | 0.03 | 5.5% | 5.0% | 0.02 |
| Epilepsy and recurrent seizures | 2.7% | 6.3% | 0.17* | 5.3% | 5.7% | 0.02 |
| Neuralgia and neuritis, unspecified | 4.7% | 4.1% | 0.03 | 4.5% | 4.3% | 0.01 |
| Alcohol abuse | 4.0% | 3.7% | 0.01 | 4.1% | 3.8% | 0.01 |
| End stage renal disease | 4.5% | 4.3% | 0.01 | 3.8% | 4.2% | 0.02 |
| Acute embolism and thrombosis of unspecified deep veins of lower extremity | 2.9% | 3.6% | 0.04 | 3.4% | 3.5% | 0.01 |
| Long term (current) use of non-steroidal anti-inflammatories (NSAID) | 1.4% | 2.6% | 0.09 | 2.0% | 2.3% | 0.02 |
| Malignant (primary) neoplasm, unspecified | 1.2% | 2.0% | 0.07 | 1.8% | 2.0% | 0.01 |
| Other postherpetic nervous system involvement | 0.6% | 0.4% | 0.03 | 0.5% | 0.5% | 0.01 |
| Ulcerative colitis, unspecified | 0.4% | 0.6% | 0.03 | 0.5% | 0.6% | 0.02 |
| Cardiovascular medications | 89.9% | 94.4% | 0.17* | 95.0% | 94.3% | 0.03 |
| Antilipemic agents | 66.0% | 74.9% | 0.19* | 75.8% | 74.5% | 0.03 |
| Insulin | 60.8% | 60.2% | 0.01 | 63.4% | 61.0% | 0.05 |
| Metformin | 51.8% | 58.0% | 0.12* | 58.6% | 57.6% | 0.02 |
| Diuretics | 50.2% | 56.0% | 0.12* | 57.4% | 56.2% | 0.02 |
| Beta blockers/related | 47.6% | 56.6% | 0.18* | 56.0% | 56.1% | 0.00 |
| Ace inhibitors | 50.8% | 54.0% | 0.06 | 55.9% | 54.2% | 0.03 |
| Aspirin | 43.1% | 51.2% | 0.16* | 51.3% | 50.6% | 0.01 |
| Hydrocodone | 36.6% | 37.3% | 0.01 | 40.9% | 38.8% | 0.04 |
| Antiarrhythmics | 31.3% | 41.7% | 0.22* | 40.4% | 41.1% | 0.02 |
| Duloxetine | 8.4% | 35.2% | 0.69* | 37.8% | 34.3% | 0.07 |
| Calcium channel blockers | 32.3% | 37.6% | 0.11* | 37.6% | 36.9% | 0.01 |
| Amitriptyline | 9.3% | 30.4% | 0.55* | 35.4% | 30.9% | 0.10 |
| Oxycodone | 28.9% | 33.4% | 0.10* | 34.7% | 34.2% | 0.01 |
| Antihypoglycemics | 27.4% | 34.9% | 0.16* | 33.7% | 34.6% | 0.02 |
| Angiotensin ii inhibitor | 22.5% | 28.8% | 0.14* | 28.5% | 28.1% | 0.01 |
| Sulfonylureas | 25.6% | 27.4% | 0.04 | 28.3% | 27.6% | 0.02 |
| Ibuprofen | 21.8% | 26.5% | 0.11* | 27.4% | 27.0% | 0.01 |
| Tramadol | 24.4% | 24.7% | 0.01 | 27.0% | 26.0% | 0.02 |
| Antihypertensives,other | 21.7% | 23.7% | 0.05 | 24.0% | 23.9% | 0.00 |
| Prednisone | 16.9% | 22.6% | 0.14* | 22.8% | 22.9% | 0.00 |
| Antianginals | 18.6% | 21.3% | 0.07 | 21.9% | 21.6% | 0.01 |
| Methylprednisolone | 15.4% | 20.9% | 0.14* | 20.3% | 20.7% | 0.01 |
| Codeine | 15.1% | 18.1% | 0.08 | 20.0% | 18.8% | 0.03 |
| Venlafaxine | 4.1% | 23.6% | 0.59* | 20.0% | 21.6% | 0.04 |
| Antipsychotics | 10.4% | 17.7% | 0.21* | 17.3% | 17.5% | 0.01 |
| Naproxen | 13.6% | 15.3% | 0.05 | 16.4% | 15.9% | 0.01 |
| Diclofenac | 9.2% | 13.9% | 0.15* | 14.3% | 14.0% | 0.01 |
| Alpha blockers/related | 10.9% | 14.3% | 0.10* | 13.4% | 14.1% | 0.02 |
| Topiramate | 2.9% | 14.5% | 0.42* | 12.6% | 14.3% | 0.05 |
| Bupropion | 6.4% | 11.8% | 0.19* | 11.6% | 11.9% | 0.01 |
| Nortriptyline | 2.9% | 10.0% | 0.29* | 10.6% | 10.0% | 0.02 |
| Thiazolidinediones | 7.8% | 9.8% | 0.07 | 10.1% | 10.0% | 0.00 |
| Hypoglycemic agents,other | 5.1% | 10.2% | 0.19* | 8.7% | 9.8% | 0.04 |
| Estrogens | 4.2% | 7.8% | 0.15* | 8.0% | 7.9% | 0.00 |
| Celecoxib | 5.5% | 6.9% | 0.06 | 7.5% | 7.3% | 0.01 |
| Capsaicin | 2.0% | 6.1% | 0.21* | 7.0% | 6.0% | 0.04 |
| Cardiovascular agents,other | 4.6% | 5.7% | 0.05 | 6.2% | 5.6% | 0.02 |
| Liraglutide | 3.6% | 6.4% | 0.13* | 6.0% | 6.3% | 0.01 |
| Sodium-glucose co-transporter 2 (sglt2) inhibitors | 3.0% | 6.3% | 0.16* | 5.1% | 5.8% | 0.03 |
| Antimigraine agents | 2.2% | 5.1% | 0.15* | 5.0% | 5.2% | 0.01 |
| Progestins | 2.7% | 4.8% | 0.11* | 4.9% | 4.8% | 0.00 |
| Exenatide | 2.7% | 4.1% | 0.08 | 4.5% | 4.3% | 0.01 |
| Carbamazepine | 0.9% | 5.2% | 0.25* | 4.1% | 5.0% | 0.04 |
| Digitalis glycosides | 2.4% | 3.2% | 0.05 | 3.0% | 3.1% | 0.01 |
| Androgens | 1.7% | 2.6% | 0.06 | 2.6% | 2.7% | 0.00 |
| Dulaglutide | 1.3% | 3.8% | 0.15* | 2.6% | 3.3% | 0.04 |
| Semaglutide | 0.3% | 1.4% | 0.13* | 0.9% | 1.5% | 0.06 |
| Peripheral vasodilators | 0.8% | 1.0% | 0.03 | 0.9% | 0.9% | 0.00 |
| Tamoxifen | 0.3% | 0.6% | 0.04 | 0.8% | 0.6% | 0.02 |
| Mineralocorticoids | 0.4% | 0.7% | 0.03 | 0.8% | 0.7% | 0.01 |
| Raloxifene | 0.3% | 0.7% | 0.06 | 0.6% | 0.7% | 0.01 |
| Tapentadol | 0.2% | 0.7% | 0.08 | 0.6% | 0.7% | 0.01 |
| Gonadotropin releasing hormone analogues | 0.3% | 0.5% | 0.04 | 0.5% | 0.5% | 0.00 |
| Antihypertensive combinations | 0.2% | 0.6% | 0.07 | 0.4% | 0.6% | 0.02 |
| Mexiletine | 0.1% | 0.8% | 0.11* | 0.4% | 0.7% | 0.05 |
| Direct renin inhibitor | 0.2% | 0.2% | 0.00 | 0.2% | 0.2% | 0.00 |
| Sclerosing agents | 0.1% | 0.2% | 0.04 | 0.2% | 0.2% | 0.01 |
| Lixisenatide | 0.0% | 0.1% | 0.03 | 0.1% | 0.1% | 0.00 |

**S4 Table:** The characteristics of the patients who were prescribed pregabalin and comparison drugs for long term before and after propensity-score matching for covariates of deep venous thrombosis. SMD – standardized mean differences. *SMD greater than 0.1, a threshold being recommended for declaring imbalance

|  | Before matching | | | After matching | | |
| --- | --- | --- | --- | --- | --- | --- |
|  | Cohort, No. (%) | | | Cohort, No. (%) | | |
|  | Pregabalin | Comparison | SMD | Pregabalin | Comparison | SMD |
| Cohort size | 8554 | 8359 |  | 3928 | 3928 |  |
| Age at Index | 58.4 ± 11.8 | 62.8 ± 12.5 | 0.37* | 61 ± 11.6 | 60.3 ± 12.9 | 0.05 |
| White | 68.0% | 72.7% | 0.10* | 71.4% | 69.8% | 0.04 |
| Female | 54.5% | 61.7% | 0.15* | 59.2% | 59.7% | 0.01 |
| Black or African American | 22.7% | 16.4% | 0.16* | 18.6% | 19.8% | 0.03 |
| Unknown Race | 7.5% | 9.5% | 0.07 | 8.6% | 8.9% | 0.01 |
| Hispanic or Latino | 8.9% | 5.9% | 0.11* | 7.2% | 7.4% | 0.01 |
| Asian | 1.0% | 0.8% | 0.03 | 0.8% | 0.9% | 0.01 |
| Hypertensive diseases | 84.0% | 87.4% | 0.10* | 85.9% | 85.6% | 0.01 |
| Overweight and obesity | 44.9% | 47.2% | 0.05 | 46.2% | 46.7% | 0.01 |
| Pure hypercholesterolemia | 23.5% | 28.6% | 0.12* | 27.3% | 27.3% | 0.00 |
| Obstructive sleep apnea (adult) (pediatric) | 23.8% | 24.5% | 0.02 | 24.1% | 25.4% | 0.03 |
| Heart failure | 18.0% | 20.0% | 0.05 | 19.3% | 19.3% | 0.00 |
| Fibromyalgia | 19.4% | 14.0% | 0.14* | 16.9% | 18.3% | 0.04 |
| Peripheral vascular disease, unspecified | 15.1% | 14.3% | 0.02 | 14.5% | 14.3% | 0.01 |
| Pain, unspecified | 12.8% | 11.7% | 0.03 | 12.1% | 13.2% | 0.03 |
| Persons with potential health hazards related to socioeconomic and psychosocial circumstances | 5.1% | 7.6% | 0.11* | 6.7% | 7.5% | 0.03 |
| Neuralgia and neuritis, unspecified | 9.9% | 4.1% | 0.23* | 6.6% | 7.1% | 0.02 |
| Tobacco use | 5.6% | 6.7% | 0.05 | 6.1% | 6.6% | 0.02 |
| Restless legs syndrome | 6.6% | 4.9% | 0.07 | 5.4% | 6.0% | 0.03 |
| Epilepsy and recurrent seizures | 3.6% | 6.3% | 0.12* | 4.6% | 4.9% | 0.02 |
| Alcohol abuse | 4.3% | 3.7% | 0.03 | 4.0% | 4.2% | 0.01 |
| End stage renal disease | 4.5% | 4.3% | 0.01 | 3.9% | 3.9% | 0.00 |
| Acute embolism and thrombosis of unspecified deep veins of lower extremity | 3.6% | 3.6% | 0.00 | 3.4% | 3.7% | 0.02 |
| Long term (current) use of non-steroidal anti-inflammatories (NSAID) | 2.1% | 2.6% | 0.03 | 2.2% | 2.4% | 0.01 |
| Malignant (primary) neoplasm, unspecified | 1.2% | 2.0% | 0.07 | 1.6% | 1.6% | 0.00 |
| Other postherpetic nervous system involvement | 1.0% | 0.4% | 0.07 | 0.6% | 0.7% | 0.01 |
| Ulcerative colitis, unspecified | 0.6% | 0.6% | 0.01 | 0.5% | 0.4% | 0.02 |
| Cardiovascular medications | 91.7% | 94.4% | 0.11* | 94.0% | 93.7% | 0.01 |
| Antilipemic agents | 70.0% | 74.9% | 0.11* | 74.2% | 74.0% | 0.01 |
| Insulin | 68.0% | 60.2% | 0.16* | 63.1% | 64.9% | 0.04 |
| Metformin | 53.6% | 58.0% | 0.09 | 57.8% | 58.2% | 0.01 |
| Diuretics | 53.5% | 56.0% | 0.05 | 56.4% | 56.7% | 0.00 |
| Beta blockers/related | 51.6% | 56.6% | 0.10* | 55.6% | 55.3% | 0.01 |
| Ace inhibitors | 53.2% | 54.0% | 0.02 | 54.3% | 53.8% | 0.01 |
| Aspirin | 48.0% | 51.2% | 0.06 | 51.5% | 51.8% | 0.01 |
| Antiarrhythmics | 42.4% | 41.7% | 0.01 | 42.6% | 44.2% | 0.03 |
| Hydrocodone | 46.9% | 37.3% | 0.20* | 42.0% | 44.9% | 0.06 |
| Calcium channel blockers | 33.2% | 37.6% | 0.09 | 36.5% | 36.3% | 0.00 |
| Oxycodone | 39.0% | 33.4% | 0.12* | 36.2% | 38.1% | 0.04 |
| Duloxetine | 21.1% | 35.2% | 0.32* | 36.0% | 35.8% | 0.00 |
| Antihypoglycemics | 34.2% | 34.9% | 0.02 | 34.3% | 35.9% | 0.03 |
| Tramadol | 32.8% | 24.7% | 0.18* | 28.8% | 30.3% | 0.03 |
| Amitriptyline | 16.7% | 30.4% | 0.33* | 28.7% | 28.4% | 0.01 |
| Angiotensin ii inhibitor | 24.9% | 28.8% | 0.09 | 28.4% | 28.1% | 0.01 |
| Ibuprofen | 27.6% | 26.5% | 0.02 | 27.4% | 29.0% | 0.04 |
| Sulfonylureas | 24.7% | 27.4% | 0.06 | 27.1% | 27.5% | 0.01 |
| Antihypertensives,other | 25.5% | 23.7% | 0.04 | 25.5% | 25.7% | 0.00 |
| Prednisone | 22.4% | 22.6% | 0.00 | 23.4% | 24.8% | 0.03 |
| Antianginals | 21.9% | 21.3% | 0.01 | 21.6% | 22.5% | 0.02 |
| Methylprednisolone | 20.0% | 20.9% | 0.02 | 20.6% | 22.5% | 0.05 |
| Codeine | 19.9% | 18.1% | 0.04 | 20.3% | 20.6% | 0.01 |
| Naproxen | 17.8% | 15.3% | 0.07 | 16.9% | 18.2% | 0.03 |
| Antipsychotics | 15.4% | 17.7% | 0.06 | 16.8% | 18.4% | 0.04 |
| Venlafaxine | 8.0% | 23.6% | 0.44* | 15.9% | 17.4% | 0.04 |
| Diclofenac | 14.7% | 13.9% | 0.02 | 14.9% | 16.0% | 0.03 |
| Alpha blockers/related | 13.4% | 14.3% | 0.02 | 14.0% | 14.4% | 0.01 |
| Topiramate | 6.1% | 14.5% | 0.28* | 11.6% | 13.5% | 0.06 |
| Bupropion | 10.5% | 11.8% | 0.04 | 11.6% | 12.6% | 0.03 |
| Nortriptyline | 6.3% | 10.0% | 0.14* | 10.6% | 10.6% | 0.00 |
| Thiazolidinediones | 9.0% | 9.8% | 0.03 | 10.2% | 10.5% | 0.01 |
| Hypoglycemic agents,other | 7.2% | 10.2% | 0.11* | 9.0% | 9.6% | 0.02 |
| Celecoxib | 9.0% | 6.9% | 0.08 | 8.2% | 9.0% | 0.03 |
| Estrogens | 5.8% | 7.8% | 0.08 | 7.3% | 8.2% | 0.04 |
| Cardiovascular agents,other | 6.5% | 5.7% | 0.03 | 6.7% | 7.2% | 0.02 |
| Capsaicin | 4.0% | 6.1% | 0.10* | 6.4% | 7.3% | 0.03 |
| Liraglutide | 5.2% | 6.4% | 0.05 | 6.1% | 6.5% | 0.02 |
| Antimigraine agents | 4.4% | 5.1% | 0.03 | 5.7% | 6.0% | 0.02 |
| Sodium-glucose co-transporter 2 (sglt2) inhibitors | 4.2% | 6.3% | 0.09 | 5.6% | 5.8% | 0.01 |
| Exenatide | 4.0% | 4.1% | 0.01 | 4.4% | 4.8% | 0.02 |
| Progestins | 3.8% | 4.8% | 0.05 | 4.2% | 5.1% | 0.04 |
| Carbamazepine | 2.1% | 5.2% | 0.17* | 3.8% | 4.6% | 0.04 |
| Androgens | 2.5% | 2.6% | 0.00 | 3.2% | 2.9% | 0.02 |
| Digitalis glycosides | 2.7% | 3.2% | 0.03 | 3.1% | 3.0% | 0.01 |
| Dulaglutide | 2.2% | 3.8% | 0.09 | 3.1% | 3.2% | 0.01 |
| Peripheral vasodilators | 1.1% | 1.0% | 0.01 | 1.2% | 0.9% | 0.03 |
| Tapentadol | 0.5% | 0.7% | 0.02 | 0.8% | 0.8% | 0.01 |
| Mineralocorticoids | 0.7% | 0.7% | 0.00 | 0.8% | 0.9% | 0.01 |
| Semaglutide | 0.4% | 1.4% | 0.10* | 0.7% | 1.0% | 0.04 |
| Tamoxifen | 0.4% | 0.6% | 0.04 | 0.6% | 0.6% | 0.01 |
| Antihypertensive combinations | 0.3% | 0.6% | 0.04 | 0.5% | 0.6% | 0.02 |
| Raloxifene | 0.3% | 0.7% | 0.06 | 0.5% | 0.5% | 0.01 |
| Gonadotropin releasing hormone analogues | 0.3% | 0.5% | 0.04 | 0.4% | 0.5% | 0.02 |
| Mexiletine | 0.2% | 0.8% | 0.09 | 0.4% | 0.6% | 0.04 |
| Direct renin inhibitor | 0.3% | 0.2% | 0.02 | 0.3% | 0.3% | 0.00 |
| Sclerosing agents | 0.1% | 0.2% | 0.03 | 0.3% | 0.3% | 0.00 |
| Lixisenatide | 0.1% | 0.1% | 0.01 | 0.3% | 0.3% | 0.00 |

**S5 Table:** The characteristics of the patients who were prescribed gabapentin and comparison drugs before and after propensity-score matching for covariates of deep venous thrombosis. SMD – standardized mean differences. *SMD greater than 0.1, a threshold being recommended for declaring imbalance

|  | Before matching | | | After matching | | |
| --- | --- | --- | --- | --- | --- | --- |
|  | Cohort, No. (%) | | | Cohort, No. (%) | | |
|  | Gabapentin | Comparison | SMD | Gabapentin | Comparison | SMD |
| Cohort size | 173052 | 22170 |  | 22029 | 22029 |  |
| Age at Index | 62.4 ± 13 | 62.8 ± 13.5 | 0.03 | 62.3 ± 12.7 | 62.8 ± 13.5 | 0.03 |
| White | 65.4% | 66.7% | 0.03 | 67.8% | 66.7% | 0.02 |
| Female | 50.0% | 54.7% | 0.09 | 57.4% | 54.6% | 0.05 |
| Black or African American | 22.9% | 19.3% | 0.09 | 19.4% | 19.3% | 0.00 |
| Unknown Race | 9.8% | 12.4% | 0.08 | 11.3% | 12.4% | 0.03 |
| Hispanic or Latino | 8.8% | 6.9% | 0.07 | 6.8% | 7.0% | 0.01 |
| Asian | 1.2% | 1.0% | 0.02 | 0.9% | 1.0% | 0.01 |
| Hypertensive diseases | 85.5% | 83.6% | 0.05 | 85.0% | 83.6% | 0.04 |
| Overweight and obesity | 41.8% | 41.4% | 0.01 | 44.4% | 41.4% | 0.06 |
| Pure hypercholesterolemia | 22.1% | 23.3% | 0.03 | 24.2% | 23.3% | 0.02 |
| Heart failure | 23.7% | 21.6% | 0.05 | 22.9% | 21.6% | 0.03 |
| Obstructive sleep apnea (adult) (pediatric) | 19.8% | 19.6% | 0.01 | 22.1% | 19.6% | 0.06 |
| Peripheral vascular disease, unspecified | 16.6% | 14.2% | 0.06 | 14.6% | 14.2% | 0.01 |
| Pain, unspecified | 12.4% | 10.7% | 0.05 | 12.2% | 10.7% | 0.05 |
| Fibromyalgia | 8.7% | 8.8% | 0.00 | 10.6% | 8.8% | 0.06 |
| Persons with potential health hazards related to socioeconomic and psychosocial circumstances | 6.2% | 6.6% | 0.01 | 7.1% | 6.6% | 0.02 |
| End stage renal disease | 8.0% | 6.4% | 0.06 | 6.6% | 6.4% | 0.01 |
| Tobacco use | 6.7% | 5.7% | 0.04 | 6.5% | 5.7% | 0.03 |
| Epilepsy and recurrent seizures | 3.1% | 3.9% | 0.04 | 4.3% | 3.9% | 0.02 |
| Restless legs syndrome | 4.2% | 3.5% | 0.04 | 4.2% | 3.5% | 0.04 |
| Acute embolism and thrombosis of unspecified deep veins of lower extremity | 3.9% | 3.3% | 0.03 | 3.6% | 3.3% | 0.02 |
| Alcohol abuse | 4.0% | 3.0% | 0.05 | 3.5% | 3.0% | 0.02 |
| Neuralgia and neuritis, unspecified | 4.1% | 2.9% | 0.06 | 3.3% | 2.9% | 0.02 |
| Long term (current) use of non-steroidal anti-inflammatories (NSAID) | 2.5% | 2.2% | 0.02 | 2.7% | 2.2% | 0.03 |
| Malignant (primary) neoplasm, unspecified | 2.2% | 1.9% | 0.02 | 2.1% | 1.9% | 0.01 |
| Ulcerative colitis, unspecified | 0.5% | 0.6% | 0.01 | 0.6% | 0.6% | 0.01 |
| Other postherpetic nervous system involvement | 0.6% | 0.3% | 0.05 | 0.3% | 0.3% | 0.01 |
| Cardiovascular medications | 91.6% | 89.9% | 0.06 | 92.7% | 90.0% | 0.10 |
| Antilipemic agents | 67.9% | 65.2% | 0.06 | 69.1% | 65.3% | 0.08 |
| Insulin | 67.1% | 61.5% | 0.12* | 65.7% | 61.6% | 0.08 |
| Beta blockers/related | 53.2% | 49.9% | 0.06 | 53.6% | 50.0% | 0.07 |
| Diuretics | 51.2% | 48.5% | 0.05 | 52.3% | 48.6% | 0.07 |
| Aspirin | 47.4% | 43.6% | 0.08 | 46.8% | 43.7% | 0.06 |
| Metformin | 47.3% | 44.5% | 0.06 | 46.7% | 44.5% | 0.04 |
| Ace inhibitors | 47.2% | 43.3% | 0.08 | 46.3% | 43.5% | 0.06 |
| Duloxetine | 9.2% | 36.2% | 0.68* | 41.9% | 36.0% | 0.12 |
| Antiarrhythmics | 40.0% | 35.2% | 0.10* | 38.4% | 35.3% | 0.06 |
| Calcium channel blockers | 35.5% | 33.3% | 0.05* | 35.2% | 33.3% | 0.04 |
| Antihypoglycemics | 37.5% | 31.9% | 0.12* | 34.8% | 32.0% | 0.06 |
| Hydrocodone | 35.0% | 28.3% | 0.14* | 32.8% | 28.5% | 0.09 |
| Oxycodone | 33.7% | 26.7% | 0.15* | 31.2% | 26.8% | 0.10* |
| Angiotensin ii inhibitor | 23.9% | 24.4% | 0.01 | 25.9% | 24.4% | 0.03 |
| Amitriptyline | 7.2% | 20.9% | 0.40* | 24.4% | 21.0% | 0.08 |
| Antihypertensives,other | 26.9% | 22.7% | 0.10 | 24.3% | 22.8% | 0.03 |
| Sulfonylureas | 22.7% | 21.0% | 0.04 | 22.3% | 21.0% | 0.03 |
| Tramadol | 24.7% | 18.3% | 0.16* | 21.8% | 18.4% | 0.09 |
| Antianginals | 20.6% | 18.3% | 0.06 | 19.9% | 18.3% | 0.04 |
| Ibuprofen | 20.1% | 16.9% | 0.08 | 18.8% | 17.0% | 0.05 |
| Prednisone | 18.0% | 15.8% | 0.06 | 18.0% | 15.9% | 0.06 |
| Methylprednisolone | 18.0% | 16.2% | 0.05 | 17.6% | 16.2% | 0.04 |
| Venlafaxine | 3.9% | 14.3% | 0.37* | 15.2% | 14.3% | 0.02 |
| Antipsychotics | 13.8% | 13.0% | 0.02 | 15.1% | 13.0% | 0.06 |
| Alpha blockers/related | 13.8% | 12.8% | 0.03 | 13.9% | 12.8% | 0.03 |
| Codeine | 13.5% | 11.5% | 0.06 | 12.9% | 11.6% | 0.04 |
| Diclofenac | 9.7% | 10.7% | 0.03 | 11.7% | 10.7% | 0.03 |
| Naproxen | 11.3% | 9.5% | 0.06 | 10.8% | 9.6% | 0.04 |
| Hypoglycemic agents,other | 7.7% | 8.9% | 0.04 | 9.7% | 8.9% | 0.03 |
| Topiramate | 2.7% | 9.0% | 0.27* | 8.9% | 9.1% | 0.01 |
| Capsaicin | 1.8% | 10.5% | 0.37* | 8.0% | 10.4% | 0.08 |
| Nortriptyline | 2.4% | 7.0% | 0.22* | 7.6% | 7.1% | 0.02 |
| Bupropion | 5.9% | 6.2% | 0.01 | 7.5% | 6.3% | 0.05 |
| Thiazolidinediones | 6.0% | 6.1% | 0.01 | 6.5% | 6.2% | 0.01 |
| Sodium-glucose co-transporter 2 (sglt2) inhibitors | 4.9% | 5.3% | 0.02 | 5.7% | 5.3% | 0.02 |
| Cardiovascular agents,other | 5.6% | 4.7% | 0.04 | 5.1% | 4.7% | 0.02 |
| Liraglutide | 4.1% | 4.6% | 0.02 | 5.0% | 4.6% | 0.02 |
| Celecoxib | 5.2% | 3.7% | 0.08 | 4.4% | 3.7% | 0.04 |
| Estrogens | 3.3% | 3.7% | 0.02 | 4.3% | 3.7% | 0.03 |
| Dulaglutide | 3.3% | 4.1% | 0.04 | 4.3% | 4.1% | 0.01 |
| Digitalis glycosides | 2.8% | 3.1% | 0.01 | 3.1% | 3.1% | 0.00 |
| Antimigraine agents | 1.9% | 2.3% | 0.03 | 2.8% | 2.4% | 0.03 |
| Exenatide | 2.2% | 2.4% | 0.01 | 2.7% | 2.4% | 0.02 |
| Progestins | 2.2% | 2.5% | 0.02 | 2.7% | 2.5% | 0.01 |
| Carbamazepine | 0.8% | 2.9% | 0.16* | 2.6% | 2.9% | 0.02 |
| Semaglutide | 1.1% | 1.5% | 0.04 | 1.6% | 1.5% | 0.01 |
| Androgens | 1.4% | 1.4% | 0.00 | 1.6% | 1.4% | 0.01 |
| Peripheral vasodilators | 1.3% | 0.8% | 0.05 | 0.9% | 0.8% | 0.01 |
| Antihypertensive combinations | 0.7% | 0.7% | 0.01 | 0.7% | 0.7% | 0.00 |
| Mineralocorticoids | 0.6% | 0.6% | 0.01 | 0.6% | 0.6% | 0.00 |
| Tapentadol | 0.2% | 0.5% | 0.05 | 0.5% | 0.5% | 0.00 |
| Mexiletine | 0.1% | 1.1% | 0.12* | 0.4% | 1.1% | 0.07 |
| Tamoxifen | 0.3% | 0.4% | 0.01 | 0.4% | 0.4% | 0.01 |
| Gonadotropin releasing hormone analogues | 0.3% | 0.4% | 0.01 | 0.4% | 0.4% | 0.01 |
| Raloxifene | 0.2% | 0.2% | 0.01 | 0.2% | 0.2% | 0.00 |
| Direct renin inhibitor | 0.2% | 0.1% | 0.00 | 0.2% | 0.1% | 0.01 |
| Sclerosing agents | 0.2% | 0.2% | 0.00 | 0.2% | 0.2% | 0.00 |
| Lixisenatide | 0.1% | 0.2% | 0.01 | 0.2% | 0.2% | 0.00 |

**S6 Table:** The characteristics of the patients who were prescribed pregabalin and comparison drugs before and after propensity-score matching for covariates of deep venous thrombosis. SMD – standardized mean differences. *SMD greater than 0.1, a threshold being recommended for declaring imbalance

|  | Before matching | | | After matching | | |
| --- | --- | --- | --- | --- | --- | --- |
|  | Cohort, No. (%) | | | Cohort, No. (%) | | |
|  | Pregabalin | Comparison | SMD | Pregabalin | Comparison | SMD |
| Cohort size | 57564 | 22170 |  | 16416 | 16416 |  |
| Age at Index | 61 ± 12.5 | 62.8 ± 13.5 | 0.14* | 62.1 ± 12.5 | 61.9 ± 13.6 | 0.01 |
| White | 66.1% | 66.7% | 0.01 | 66.8% | 66.3% | 0.01 |
| Female | 52.2% | 54.7% | 0.05 | 55.2% | 54.2% | 0.02 |
| Black or African American | 22.1% | 19.3% | 0.07 | 20.0% | 20.2% | 0.00 |
| Unknown Race | 10.0% | 12.4% | 0.08 | 11.6% | 11.8% | 0.01 |
| Hispanic or Latino | 8.2% | 6.9% | 0.05 | 7.5% | 7.3% | 0.01 |
| Asian | 1.0% | 1.0% | 0.00 | 1.0% | 1.0% | 0.00 |
| Hypertensive diseases | 85.9% | 83.6% | 0.06 | 84.3% | 83.9% | 0.01 |
| Overweight and obesity | 48.3% | 41.4% | 0.14* | 43.8% | 43.8% | 0.00 |
| Pure hypercholesterolemia | 24.5% | 23.3% | 0.03 | 24.1% | 23.8% | 0.01 |
| Heart failure | 25.1% | 21.6% | 0.08 | 22.3% | 22.6% | 0.01 |
| Obstructive sleep apnea (adult) (pediatric) | 26.1% | 19.6% | 0.16* | 21.5% | 21.3% | 0.00 |
| Peripheral vascular disease, unspecified | 18.7% | 14.2% | 0.12* | 15.3% | 15.3% | 0.00 |
| Pain, unspecified | 18.7% | 10.7% | 0.23* | 12.9% | 12.8% | 0.00 |
| Fibromyalgia | 15.0% | 8.8% | 0.19* | 11.3% | 10.8% | 0.02 |
| Persons with potential health hazards related to socioeconomic and psychosocial circumstances | 8.6% | 6.6% | 0.08 | 7.6% | 7.5% | 0.00 |
| Tobacco use | 9.9% | 5.7% | 0.16* | 6.9% | 6.9% | 0.00 |
| End stage renal disease | 8.5% | 6.4% | 0.08 | 6.8% | 6.8% | 0.00 |
| Restless legs syndrome | 6.7% | 3.5% | 0.14* | 4.3% | 4.3% | 0.00 |
| Epilepsy and recurrent seizures | 3.9% | 3.9% | 0.00 | 4.2% | 3.9% | 0.02 |
| Neuralgia and neuritis, unspecified | 9.6% | 2.9% | 0.28* | 4.0% | 3.9% | 0.01 |
| Acute embolism and thrombosis of unspecified deep veins of lower extremity | 5.5% | 3.3% | 0.11* | 4.0% | 3.9% | 0.01 |
| Alcohol abuse | 5.1% | 3.0% | 0.11* | 3.5% | 3.6% | 0.00 |
| Long term (current) use of non-steroidal anti-inflammatories (NSAID) | 4.0% | 2.2% | 0.10* | 2.7% | 2.7% | 0.00 |
| Malignant (primary) neoplasm, unspecified | 2.8% | 1.9% | 0.06 | 2.3% | 2.2% | 0.01 |
| Ulcerative colitis, unspecified | 0.6% | 0.6% | 0.01 | 0.6% | 0.6% | 0.00 |
| Other postherpetic nervous system involvement | 1.2% | 0.3% | 0.11* | 0.4% | 0.3% | 0.01 |
| Cardiovascular medications | 93.8% | 89.9% | 0.14* | 91.9% | 91.5% | 0.01 |
| Antilipemic agents | 73.0% | 65.2% | 0.17* | 68.6% | 67.9% | 0.01 |
| Insulin | 72.9% | 61.5% | 0.24* | 65.4% | 65.1% | 0.01 |
| Beta blockers/related | 58.5% | 49.9% | 0.17* | 52.8% | 52.7% | 0.00 |
| Diuretics | 57.2% | 48.5% | 0.18* | 51.6% | 51.4% | 0.00 |
| Metformin | 52.4% | 44.5% | 0.16* | 47.7% | 47.4% | 0.01 |
| Aspirin | 54.5% | 43.6% | 0.22* | 47.3% | 47.4% | 0.00 |
| Ace inhibitors | 52.0% | 43.3% | 0.17* | 46.7% | 46.1% | 0.01 |
| Antiarrhythmics | 51.8% | 35.2% | 0.34* | 40.1% | 40.6% | 0.01 |
| Duloxetine | 21.3% | 36.2% | 0.33* | 38.1% | 34.6% | 0.07 |
| Calcium channel blockers | 39.2% | 33.3% | 0.12* | 35.7% | 35.2% | 0.01 |
| Antihypoglycemics | 44.9% | 31.9% | 0.27* | 35.5% | 35.6% | 0.00 |
| Hydrocodone | 46.1% | 28.3% | 0.37* | 34.0% | 33.8% | 0.00 |
| Oxycodone | 46.5% | 26.7% | 0.42* | 32.2% | 32.7% | 0.01 |
| Angiotensin ii inhibitor | 27.5% | 24.4% | 0.07 | 26.0% | 25.7% | 0.01 |
| Antihypertensives,other | 31.9% | 22.7% | 0.21* | 25.2% | 25.4% | 0.00 |
| Tramadol | 36.5% | 18.3% | 0.42* | 23.3% | 23.0% | 0.01 |
| Amitriptyline | 14.4% | 20.9% | 0.17* | 23.2% | 21.8% | 0.03 |
| Sulfonylureas | 23.9% | 21.0% | 0.07 | 22.2% | 22.1% | 0.00 |
| Ibuprofen | 28.3% | 16.9% | 0.27* | 21.0% | 20.3% | 0.02 |
| Antianginals | 25.2% | 18.3% | 0.17* | 20.4% | 20.5% | 0.00 |
| Prednisone | 26.1% | 15.8% | 0.25* | 18.8% | 18.6% | 0.01 |
| Methylprednisolone | 24.8% | 16.2% | 0.22* | 18.8% | 18.4% | 0.01 |
| Antipsychotics | 19.2% | 13.0% | 0.17* | 14.5% | 14.7% | 0.00 |
| Codeine | 19.3% | 11.5% | 0.22* | 14.3% | 13.8% | 0.01 |
| Alpha blockers/related | 17.1% | 12.8% | 0.12* | 14.0% | 14.1% | 0.00 |
| Venlafaxine | 7.5% | 14.3% | 0.22* | 13.5% | 13.2% | 0.01 |
| Diclofenac | 17.2% | 10.7% | 0.19* | 12.6% | 12.3% | 0.01 |
| Naproxen | 17.2% | 9.5% | 0.23* | 12.1% | 11.7% | 0.01 |
| Hypoglycemic agents,other | 11.0% | 8.9% | 0.07 | 10.2% | 9.8% | 0.01 |
| Topiramate | 5.8% | 9.0% | 0.12* | 9.8% | 10.0% | 0.01 |
| Capsaicin | 4.3% | 10.5% | 0.24* | 8.2% | 9.9% | 0.06 |
| Nortriptyline | 5.5% | 7.0% | 0.07 | 8.0% | 8.1% | 0.00 |
| Bupropion | 9.5% | 6.2% | 0.12* | 7.9% | 7.4% | 0.02 |
| Thiazolidinediones | 7.5% | 6.1% | 0.05 | 6.8% | 6.6% | 0.01 |
| Sodium-glucose co-transporter 2 (sglt2) inhibitors | 6.3% | 5.3% | 0.04 | 5.8% | 5.6% | 0.01 |
| Cardiovascular agents,other | 8.6% | 4.7% | 0.16* | 5.5% | 5.7% | 0.01 |
| Liraglutide | 6.3% | 4.6% | 0.08 | 5.4% | 5.2% | 0.01 |
| Celecoxib | 10.7% | 3.7% | 0.28* | 4.8% | 4.8% | 0.00 |
| Dulaglutide | 4.7% | 4.1% | 0.03 | 4.6% | 4.4% | 0.01 |
| Estrogens | 4.9% | 3.7% | 0.06 | 4.4% | 4.2% | 0.01 |
| Antimigraine agents | 4.0% | 2.3% | 0.09 | 3.3% | 3.0% | 0.01 |
| Digitalis glycosides | 3.0% | 3.1% | 0.00 | 3.1% | 3.1% | 0.00 |
| Progestins | 3.5% | 2.5% | 0.06 | 3.0% | 2.8% | 0.01 |
| Carbamazepine | 1.8% | 2.9% | 0.07 | 2.9% | 3.2% | 0.02 |
| Exenatide | 3.2% | 2.4% | 0.05 | 2.8% | 2.7% | 0.01 |
| Semaglutide | 1.6% | 1.5% | 0.01 | 1.7% | 1.6% | 0.01 |
| Androgens | 2.1% | 1.4% | 0.05 | 1.6% | 1.6% | 0.00 |
| Peripheral vasodilators | 1.6% | 0.8% | 0.08 | 0.9% | 1.0% | 0.01 |
| Antihypertensive combinations | 0.8% | 0.7% | 0.01 | 0.7% | 0.8% | 0.01 |
| Mineralocorticoids | 1.0% | 0.6% | 0.05 | 0.7% | 0.7% | 0.00 |
| Tapentadol | 0.7% | 0.5% | 0.03 | 0.7% | 0.6% | 0.01 |
| Mexiletine | 0.2% | 1.1% | 0.11* | 0.5% | 1.1% | 0.08 |
| Gonadotropin releasing hormone analogues | 0.4% | 0.4% | 0.01 | 0.4% | 0.5% | 0.00 |
| Tamoxifen | 0.4% | 0.4% | 0.00 | 0.4% | 0.4% | 0.00 |
| Sclerosing agents | 0.3% | 0.2% | 0.03 | 0.2% | 0.2% | 0.00 |
| Raloxifene | 0.3% | 0.2% | 0.01 | 0.2% | 0.2% | 0.00 |
| Direct renin inhibitor | 0.2% | 0.1% | 0.01 | 0.2% | 0.2% | 0.01 |
| Lixisenatide | 0.2% | 0.2% | 0.00 | 0.2% | 0.2% | 0.01 |
